# Robotic-assisted thoracic surgery following neoadjuvant chemoimmunotherapy in patients with stage III non-small cell lung cancer: A real-world prospective cohort study

**DOI:** 10.3389/fonc.2022.969545

**Published:** 2022-08-04

**Authors:** Yang Gao, Juan Jiang, Desheng Xiao, Yanwu Zhou, Yufan Chen, Huaping Yang, Lijing Wang, Jun Zeng, Baimei He, Ruoxi He, Min Li, Zhaoqian Liu

**Affiliations:** ^1^ Department of Thoracic Surgery, Xiangya Hospital, Central South University, Changsha, China; ^2^ Department of Clinical Pharmacology, Hunan Key Laboratory of Pharmacogenetics, Xiangya Hospital, Central South University, Changsha, China; ^3^ Hunan Engineering Research Center for Pulmonary Nodules Precise Diagnosis & Treatment, Changsha, China; ^4^ Xiangya Lung Cancer Center, Xiangya Hospital, Central South University, Changsha, China; ^5^ Department of Respiratory Medicine, National Key Clinical Specialty, Branch of National Clinical Research Center for Respiratory Disease, Xiangya Hospital, Central South University, Changsha, China; ^6^ Clinical Research Center for Respiratory Diseases in Hunan Province, Changsha, China; ^7^ Hunan Engineering Research Center for Intelligent Diagnosis and Treatment of Respiratory Disease, Changsha, China; ^8^ Department of Pathology, Xiangya Hospital, School of Basic Medicine, Central South University, Changsha, China; ^9^ Department of Geriatric Medicine, Xiangya Hospital, Central South University, Changsha, China; ^10^ National Clinical Research Center for Geriatric Disorders, Xiangya Hospital, Central South University, Changsha, China

**Keywords:** non-small cell lung cancer, neoadjuvant therapy, chemoimmunotherapy, robotic-assisted thoracic surgery, neoadjuvant immunotherapy

## Abstract

**Objective:**

Stage III non-small cell lung cancer (NSCLC) is a heterogeneous group of diseases. For this subset of patients, clinical management is still under debate and prognosis remains poor so far. In the present study, we aimed to evaluate the feasibility and safety of robotic-assisted thoracic surgery after neoadjuvant chemoimmunotherapy in stage III NSCLC.

**Methods:**

A real-world prospective cohort study was performed in a single-center setting from April 2021 to May 2022. Patients who were diagnosed with resectable or potentially resectable stage IIIA–B NSCLC and received neoadjuvant chemoimmunotherapy followed by robotic-assisted thoracic surgery were enrolled. Pathological response to neoadjuvant chemoimmunotherapy, treatment-related adverse events, and surgical outcomes of these patients were evaluated.

**Results:**

A total of 44 patients who underwent robotic-assisted thoracic surgery after three doses of neoadjuvant chemoimmunotherapy were included in this study. Of these, 36 of 44 (81.8%) patients had a major pathological response, and 26 (59.1%) had a pathological complete response based on pathological examination of surgical specimen. Eight patients (18.2%) suffered grade 3 treatment-related adverse events, including neutropenia (n = 4), increased aminotransferases (n = 3), anemia (n = 1), and cutaneous capillary endothelial proliferation (n = 1). Robotic-assisted thoracic surgery was performed subsequently, and R0 resection was achieved in all patients. Only two (4.5%) patients required conversion to thoracotomy. Surgical complications occurred in five (11.4%) patients, including air leak (n = 3), chylothorax (n = 2), and surgical site infection (n = 1). There was no re-surgery or postoperative mortality within 90 days.

**Conclusion:**

Robotic-assisted thoracic surgery following neoadjuvant chemoimmunotherapy showed good feasibility and safety in stage III NSCLC. It was not associated with unexpected perioperative morbidity or mortality and may be a promising therapeutic option in stage III NSCLC. These results need further confirmation by more large-scale clinical trials.

## Introduction

NSCLC accounts for 80%–85% of all lung cancers worldwide and has become the top killer among cancers (1). Approximately 30% of patients with NSCLC are diagnosed with stage III disease, which represents a potentially curable disease (2). However, clinical prognosis of this subset of patients remains poor with a 5-year overall survival ranging from 13% to 36% (3).

Stage III NSCLC is a heterogeneous group of diseases with varying tumor and nodal statuses, treatment options, and prognosis. Multidisciplinary cooperation and multimodality treatments including radiotherapy, chemotherapy, immunotherapy, and surgical resection are required when dealing with patients with stage III NSCLC (4). Currently, surgical resection with adjuvant therapy is the first option for patients with resectable stage III NSCLC, and surgery plus neoadjuvant therapy is recommended in potentially resectable diseases, while radical concurrent chemoradiotherapy is recommended for those with unresectable diseases. Over the past decades, numerous studies have been conducted using chemotherapeutic agents, radiotherapy, and immune checkpoint inhibitors in stage III NSCLC. Despite survival advantages of neoadjuvant chemotherapy have been confirmed, the 5-year overall survival rate is slightly increased by 5% for patients with stage III NSCLC. In recent years, neoadjuvant immunotherapy with immune checkpoint inhibitors, combined with chemotherapy or not, has shed new light to this subpopulation (5). However, it also brings challenges including immune-related adverse events, surgical delay, increased surgical complexity, and conversion to thoracotomy. Therefore, there is an urgent need for exploring more effective multimodality therapeutic strategies to improve prognosis of patients with stage III NSCLC.

Robotic-assisted thoracic surgery (RATS) is an optional minimally invasive surgical approach for patients with NSCLC. Compared with thoracotomy, RATS offers numerous benefits, including reduced surgical trauma, milder postoperative pain, and less complications. Furthermore, RATS had several advantages over video-assisted thoracoscopic surgery (VATS). The robotic system provided improved three-dimensional vision and advanced instruments with more degrees of motion freedom, higher resolution, and better ergonomics (6). It has been reported that robotic-assisted surgeries were associated with reductions in mortality, length of stay, and complication rates when compared with thoracotomy and VATS (7, 8). However, it is unknown whether RATS might have any potential benefits in pulmonary resection of stage III NSCLC after neoadjuvant chemoimmunotherapy, which usually represents more complex thoracic surgical procedures.

Herein, we investigated the efficacy and safety of a therapeutic strategy combining neoadjuvant chemoimmunotherapy with RATS, through analyzing the real-world data of 44 patients with stage IIIA-B NSCLC who underwent RATS following neoadjuvant chemoimmunotherapy at Xiangya Hospital.

## Methods

### Study design and participants

This is a real-world prospective cohort study conducted at a tertiary hospital in China from 1 April 2021 to 31 May 2022. The inclusion criteria of patients were listed as follows: (1) adult NSCLC patients, which was histologically confirmed in tissue; (2) stages IIIA/B eligible for surgery which were evaluated by comprehensive imaging examinations and lung function test; (3) no systemic cancer therapy was received; (4) ECOG performance status score ≤2. Patients who met any of the following criteria were excluded: (1) <18 years old; (2) EGFR or ALK aberrations positive; (3) immunodeficiency diseases, interstitial lung diseases, active hepatitis B, active tuberculosis, and current systemic immunosuppressive therapy with either corticosteroids (>10 mg daily prednisolone equivalent) or other immunosuppressive agents; (4) concurrent solid or hematological malignancies; (5) any previous medical treatment with immune checkpoint inhibitors. A total of 44 treatment-naive patients were diagnosed as resectable or potentially resectable stage III NSCLC by a multidisciplinary team at Xiangya Hospital, received neoadjuvant chemoimmunotherapy, and underwent RATS. This work has been reported in line with the STROCSS criteria (9). This study was registered in the Chinese Clinical Trial Registry (registration number: ChiCTR2200057840) and approved by the Institutional Review Board and Ethics Committee of Central South University (202104002).

### Therapy procedures

Patients received three cycles of immune checkpoint inhibitors and platinum-based doublet chemotherapy as neoadjuvant treatment before surgical resection. All the drugs were administrated intravenously on day 1 of each 21-day treatment cycle. Before each treatment cycle, laboratory blood tests were routinely performed to monitor blood cell counts and biochemical parameters. After the completion of neoadjuvant chemoimmunotherapy, patients underwent a standard preoperative staging workup to assess the feasibility of surgical resection, including contrast-enhanced computed tomography (CT) scan of the chest, (18)F-fluorodeoxyglucose positron emission tomography/CT scan, brain imaging with magnetic resonance imaging or CT, and bronchoscopy examination before surgery. Subsequently, resection of the primary tumor and lymph nodes was completed by using the da Vinci surgical system (Intuitive Surgical, California, USA) according to standard institutional procedures.

### Pathological response assessments

Pathological responses of patients were used to evaluate the efficacy of neoadjuvant chemoimmunotherapy. Surgical samples of primary tumor from lung and lymph nodes were examined in the Department of Pathology and staged according to the criteria of the American Joint Committee on Cancer (the eighth edition). Percentages of residual viable tumor cells were determined by routine hematoxylin and eosin staining. Major pathological response (MPR) was defined as no more than 10% viable tumor cells remaining in the primary tumor on postoperative pathologic review. Incomplete pathological response (IPR) was defined as the presence of more than 10% viable tumor cells in the primary tumor. Pathological complete response (pCR) was defined as no viable tumor cells remaining in the resected lung cancer specimen and all sampled regional lymph nodes (10–12).

### Clinical data collection

Data of demographic information, clinical characteristics, histology subtypes, neoadjuvant treatment regimens, pathological responses, surgical details, and perioperative outcomes were extracted from medical records of patients. Postoperative 30- and 90-day mortality was obtained by routine monthly follow-up after surgery. Adverse events were assessed according to the National Cancer Institute Common Terminology Criteria for Adverse Events (CTCAE, V5.0). Surgical complications were defined according to the Society of Thoracic Surgeons database criteria. For all clinical data in this study, continuous variables were expressed as median and interquartile ranges. Categorical variables were expressed as numbers and percentages.

## Results

### Baseline characteristics of patients

As shown in **Table 1**, a total of 44 patients diagnosed as stage III NSCLC were included in this study. Their ages ranged from 35 to 70 years (median age: 61.5 years). Among these patients, 33 (75.0%) were men and 33 (75.0%) were current or former smokers. Forty-one of 44 (93.2%) patients had ECOG performance status score ≤1. Preoperative FEV1% predicted ranged from 60% to 100% (median FEV1% predicted: 85%). Overall, 61.4% of them were confirmed as stage IIIA and 38.6% as stage IIIB at baseline. A total of 33 (75.0%) patients were diagnosed as squamous cell carcinoma, while 10 (22.7%) were adenocarcinoma and one (2.3%) was adenosquamous carcinoma by preoperative tumor biopsy. There were six kinds of PD-1 inhibitors used for neoadjuvant therapy, including nivolumab (n = 20), camrelizumab (n = 8), toripalimab (n = 6), tislelizumab (n = 4), sintilimab (n = 4), and pembrolizumab (n = 2). Detailed baseline characteristics of each patient are shown in **Supplementary Table 1**.

**Table 1:** Baseline characteristics of patients and pathological responses to neoadjuvant chemoimmunotherapy.

Characteristics	Median (IQR) or n (%)
Age, years Male Smoking history Non-smoker Former or current smoker ECOG PS score 0 1 2 FEV1% predicted Clinical stage IIIA IIIB Histologic subtype Squamous cell carcinoma Adenocarcinoma Others Neoadjuvant chemoimmunotherapy ICI types Nivolumab Camrelizumab Toripalimab Tislelizumab Sintilimab Pembrolizumab Chemotherapy regimens TC TP PC TL Pathological response MPR pCR	61.5 (54-65) 33 (75.0) 11 (25.0) 33 (75.0) 27 (61.4) 14 (31.8) 3 (6.8) 85% (75-90%) 27 (61.4) 17 (38.6) 33 (75.0) 10 (22.7) 1 (2.3) 20 (45.5) 8 (18.2) 6 (13.6) 4 (9.1) 4 (9.1) 2 (4.5) 31 (70.5) 6 (13.6) 5 (11.4) 2 (4.5) 36 (81.8) 26 (59.1)

PS, performance status; Scc, squamous cell carcinoma; Ade, adenocarcinoma; ICI, immune checkpoint inhibitors; TC, paclitaxel plus carboplatin; TP, paclitaxel plus cisplatin; PC, pemetrexed plus carboplatin; TL, paclitaxel plus lobaplatin; pCR, pathological complete response; MPR, major pathological response; IQR, interquartile range which describes the middle 50% of values when ordered from lowest to highest; FEV1, forced expiratory volume in one second.

### Pathological response to neoadjuvant chemoimmunotherapy and treatment-related adverse events

**Figure 1:**
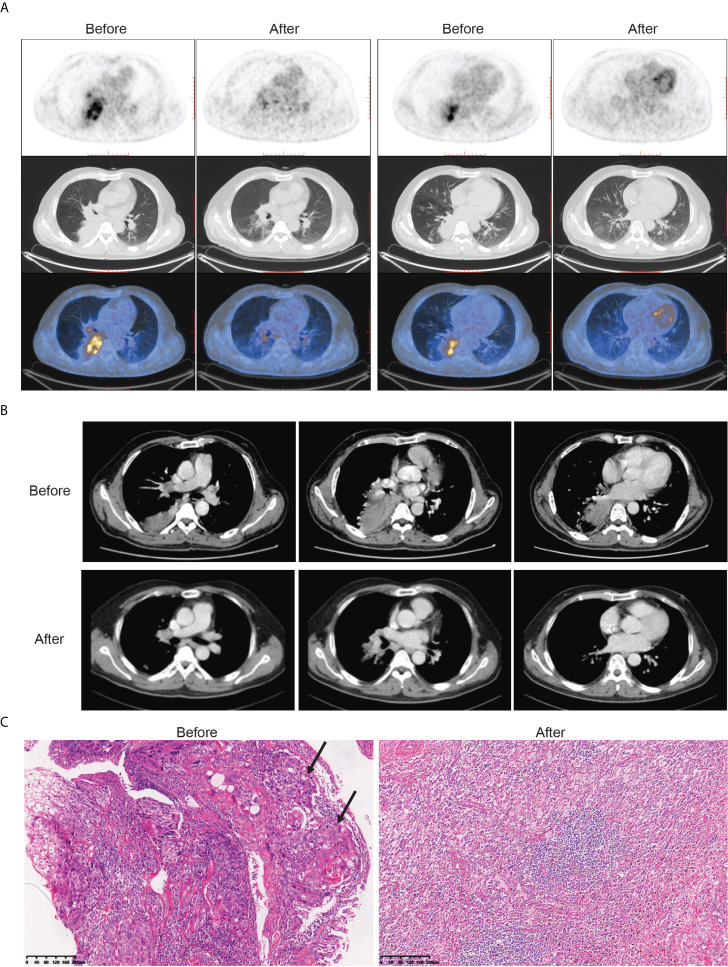
Radiological findings, and histological images in one patient (case 7) with stage III NSCLC who underwent robotic-assisted thoracic surgery following neoadjuvant chemoimmunotherapy. Case 7 had a pathological complete response to neoadjuvant chemoimmunotherapy. **(A)** Representative (18)F-fluorodeoxyglucose positron emission tomography/computed tomography before and after neoadjuvant chemoimmunotherapy. **(B)** Representative chest computed tomography imaging before and after neoadjuvant chemoimmunotherapy. **(C)** Histological examinations of pretreatment tumor biopsy and posttreatment resected tumor specimen by hematoxylin and eosin staining. Poorly differentiated tumor cells (black arrow) were observed before neoadjuvant chemoimmunotherapy, which was replaced by fibrotic, elastostatic, and necrotic tissue mixed with inflammatory cell infiltration afterward. Scale bar = 200 μm.

**Figure 2:**
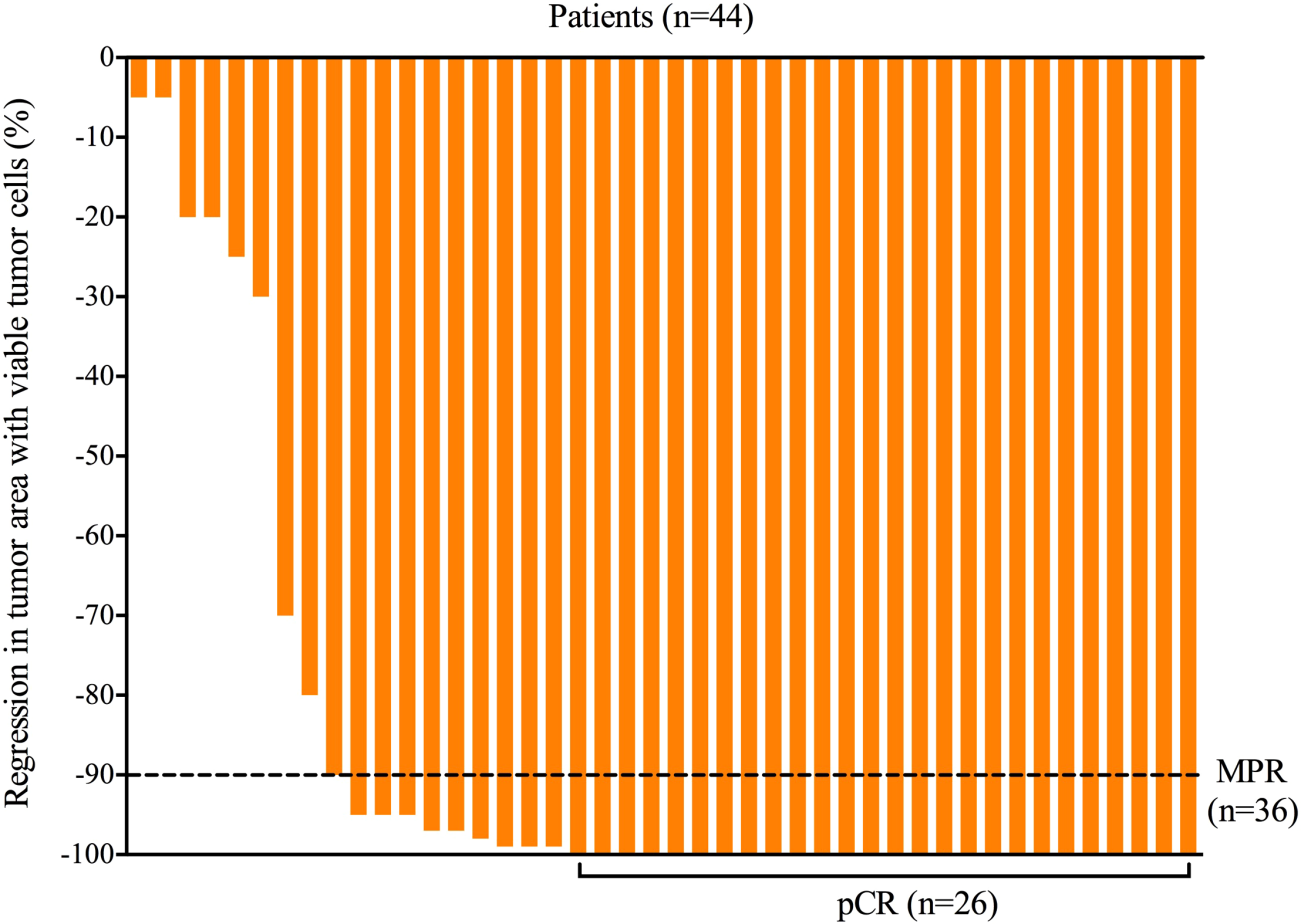
Regression in tumor area with viable tumor cells in surgical specimen of all patients after neoadjuvant chemoimmunotherapy. MPR, major pathological response; pCR, pathological complete response.

All patients received three cycles of neoadjuvant immunotherapy plus platinum-based doublet chemotherapy before surgery. In total, 36 (81.8%) of 44 patients who underwent surgery had an MPR and 26 (59.1%) had a pCR (**Table 1**). Representative radiological and histological images of case 7, who achieved pCR after neoadjuvant chemoimmunotherapy, are shown in **Figure 1**. Regression in the tumor area with viable tumor cells in surgical specimen of these patients is summarized in **Figure 2**. Overall, 83% of patients suffered treatment-related adverse events of any grade after neoadjuvant chemoimmunotherapy (**Table 2**). Only eight patients (18.2%) presented grade 3 adverse events, including neutropenia (n = 4), increased aminotransferases (n = 3), anemia (n = 1), and cutaneous capillary endothelial proliferation (n = 1). No grade 4 or 5 adverse events were observed. The most common treatment-related adverse events included neutropenia (29.6%), increased aminotransferases (20.4%), anemia (18.2%), neurotoxic effects (18.2%), rash (13.6%), fatigue (11.4%), and decreased appetite (11.4%).

**Table 2:** Treatment-related adverse events of neoadjuvant chemoimmunotherapy.

Adverse events, n (%)	Grade 1-2	Grade 3
Neutropenia Increased aminotransferases Anemia Neurotoxic effects Rash Fatigue Decreased appetite Arthralgia Thrombocytopenia Alopecia Hiccup Constipation Hyperthyroidism Nausea Oral ulcer Hypothyroidism Hyperglycemia Headache Cutaneous capillary endothelial proliferation	9 (20.5) 6 (13.6) 7 (15.9) 8 (18.2) 6 (13.6) 5 (11.4) 5 (11.4) 4 (9.1) 4 (9.1) 3 (6.8) 3 (6.8) 3 (6.8) 3 (6.8) 2 (4.5) 2 (4.5) 1 (2.3) 1 (2.3) 1 (2.3) 1 (2.3)	4 (9.1) 3 (6.8) 1(2.3) 0 0 0 0 0 0 0 0 0 0 0 0 0 0 0 1 (2.3)

### Surgical outcomes of patients undergoing RATS after neoadjuvant chemoimmunotherapy

**Figure 3:**
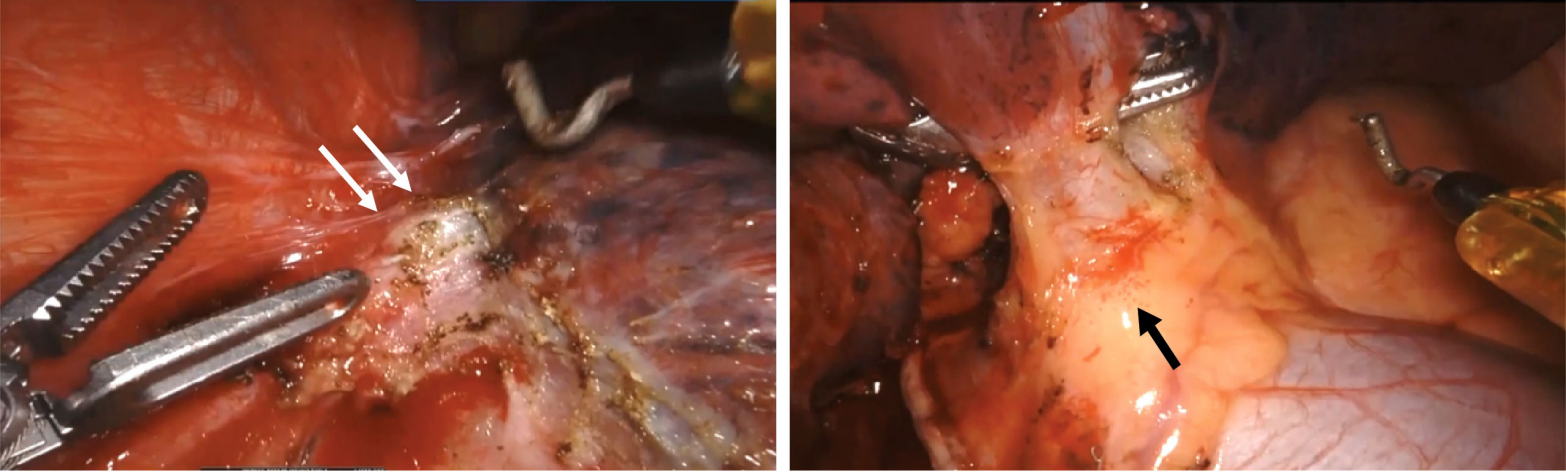
Representative intraoperative views of one patient (case 7) during robotic-assisted thoracic surgery. It showed adhesion and fibrosis (white arrow), edema, and microbleeds (black arrow) in the chest after neoadjuvant chemoimmunotherapy.

Surgical outcomes are summarized in **Table 3**. All 44 patients underwent RATS after neoadjuvant chemoimmunotherapy. R0 resection was performed in all patients, and neoadjuvant chemoimmunotherapy did not delay planned surgery. Lobectomy, bilobectomy, sleeve lobectomy, and pneumonectomy were performed in 39, two, two, and one patient, respectively. Adhesion, fibrosis, edema, and microbleeds in the chest were commonly observed during surgery (**Figure 3**). A surgical video for case 7 was attached as supplementary materials to show more details of RATS procedures. The median surgical time of these patients was 191 min (interquartile ranges: 150–235 min). The median estimated blood loss was 100 ml (interquartile ranges: 50–150 ml). Only two (4.5%) required conversion to thoracotomy. Surgical complications occurred in five (11.4%) patients, including air leak (n = 3), chylothorax (n = 2), and surgical site infection (n = 1). The median postoperative length of stay was 6.5 days. No patient died within 30 or 90 days after surgery. Detailed surgical outcomes of each patient are shown in **Supplementary Table 2**.

**Table 3:** Surgical outcomes of patients undergoing robotic-assisted resection after neoadjuvant chemoimmunotherapy.

Outcomes	Median (IQR) or n (%)
R0 resection Incidence of surgical delay Extent of resection Lobectomy Sleeve lobectomy Bilobectomy Pneumonectomy Surgical time (min) Estimated blood loss (mL) Conversion to thoracotomy Intraoperative transfusion Re-surgery Surgical complications Air leak Chylothorax Surgical site infection Postoperative length of stay (days) 30-day mortality 90-day mortality	44 (100) 0 39 (88.6) 2 (4.5) 2 (4.5) 1 (2.3) 191 (150-235) 100 (50-150) 2 (4.5) 3 (6.8) 0 5 (11.4) 3 (6.8) 2 (4.5) 1 (2.3) 6.5 (5-8) 0 0

IQR, interquartile range which describes the middle 50% of values when ordered from lowest to highest.

## Discussion

In this study, we investigated the feasibility and safety of RATS after neoadjuvant chemoimmunotherapy in stage III NSCLC, which has not been well defined in previous studies. Our data support that neoadjuvant chemoimmunotherapy followed by RATS may be a promising therapeutic approach with a high pCR rate and low incidence of conversions and surgical complications for patients with stage III NSCLC.

Neoadjuvant therapy is an effective approach for patients with stage III NSCLC to increase resectability and extend survival (13). While meta-analyses of neoadjuvant chemotherapy revealed a significant survival advantage (HR = 0.87, P = 0.007) over surgery alone, only 22% of patients with stage I–IIIA NSCLC who received neoadjuvant chemotherapy achieved MPR, and 4% achieved pCR (10, 14). Encouragingly, neoadjuvant immunotherapy with immune checkpoint inhibitors has shed new light to resectable NSCLC, with an MPR rate ranging from 21% and 45%, acceptable toxicity, no delay in surgery, and no increase in operative mortality (15–17, 18). Recently, the efficacy and safety of neoadjuvant chemoimmunotherapy has been demonstrated by a series of clinical trials. In patients with resectable NSCLC in stages I–III, neoadjuvant atezolizumab plus chemotherapy led to an MPR rate of 50% and a pCR rate of 21.4% without surgical delay. Downstaging of nodal status was confirmed in 69% of patients with N2 at baseline after neoadjuvant therapy (19). In the NADIM trial (11), neoadjuvant nivolumab and chemotherapy were given to patients with stage IIIA resectable NSCLC. Remarkably, the results showed that 85% of patients had MPR, 71% had pCR, and 90% of patients achieved pathological downstaging of the clinical disease stage before surgery, with no surgical delay reported. The overall survival at 24 months was 100% in patients who achieved an MPR or pCR after neoadjuvant chemoimmunotherapy, and progression-free survival in patients with a pCR was significantly higher than that in patients with an IPR or MPR. Furthermore, updated research data from the CheckMate 816 trial (an ongoing phase 3 multicenter randomized controlled trial) strongly demonstrated the advantages of neoadjuvant chemoimmunotherapy over chemotherapy alone in NSCLC (20). Besides, several small-scale clinical studies also supported the efficacy and safety of neoadjuvant chemoimmunotherapy in a Chinese population with resectable NSCLC (21–23). Therefore, preoperative immunotherapy combined with chemotherapy is believed to represent a promising therapeutic option to increase resectability and improve the prognosis of NSCLC.

In this study, we concentrated on exploring an effective and safe therapeutic strategy for patients in stage III NSCLC, which encompasses a variety of local invasion and nodal involvement. Till today, clinical management of stage III NSCLC is still under debate. Our data consistently showed that neoadjuvant chemoimmunotherapy achieved a high MPR rate of 81.8% and a pCR rate of 59.1% in patients who were diagnosed with stage IIIA–B NSCLC and underwent surgery, with well-tolerable toxicity. Moreover, combination of immunotherapy and chemotherapy did not cause surgical delay despite increasing incidence of grade 1–2 treatment-related adverse events. Together with results from previous clinical trials, our data consistently support that neoadjuvant chemoimmunotherapy outperforms neoadjuvant chemotherapy or immunotherapy alone in pathological response without increasing surgical delay or severe adverse events, which may further improve the long-term prognosis of stage III NSCLC. It is noteworthy to mention that the pathological response to neoadjuvant chemoimmunotherapy in the NADIM trial (11) and our study, which focused on stage III NSCLC, was markedly better when compared with the NCT02716038 and CheckMate 816 trials which enrolled patients in stage I–III diseases [15, 16]. More large-scale clinical trials are required to confirm this phenomenon and investigate the potential mechanisms.

As an option for minimally invasive thoracic surgery, RATS has shown at least comparable perioperative outcomes to those achieved by VATS in NSCLC (7, 8). Besides, a recent study further showed that robotic-assisted thoracic surgery was more cost-effective than open thoracotomy (24). Previously, the feasibility and safety of RATS in NSCLC have been demonstrated, especially for patients with a pathologic N2 disease (25). However, the safety and feasibility of RATS after neoadjuvant chemoimmunotherapy in stage III NSCLC remain unclear. Thus, RATS was attempted in this study for patients with stage III NSCLC after three doses of neoadjuvant chemoimmunotherapy in order to combine the advantages of these two therapeutics. R0 resection was achieved in all 44 patients, and the median surgical time of RATS (191 min) was similar to other surgical approaches (184 min) as reported in the CheckMate 816 trial (26). Only five (11.4%) patients had surgical complications, and no re-surgery or postoperative 90-day death event occurred. Our data support that RATS following neoadjuvant chemoimmunotherapy is safe and feasible in stage III NSCLC.

The risk of conversion to thoracotomy due to technical difficulties and serious intraoperative complications has been worrying for patients receiving neoadjuvant chemoimmunotherapy, especially for those with stage III NSCLC. In recent years, increasing amounts of studies have reported that neoadjuvant immunotherapy can cause significant adhesions, edema, and fibrosis in the chest that may increase surgical complexity and risk of conversion, which is particularly the case in patients with a significant treatment response (13). In 2017, Chaft and his colleagues (27) firstly described that dense fibrosis occurred after neoadjuvant T-cell checkpoint inhibitors in a series of NSCLC patients. In the TOP1201 trial, 12 patients with NSCLC were treated with preoperative chemotherapy and ipilimumab followed by VATS, and three of 12 (25%) converted VATS to open thoracotomy (28). Among 13 patients who attempted VATS or the robotic approach after three cycles of neoadjuvant nivolumab in the CheckMate159 trial (29), seven (54%) required conversion to thoracotomy, and the conversion rate was even higher (71%) in stage IIB/IIIA cases. Encouragingly, in the present study, only two out of 44 (4.5%) patients required conversion to thoracotomy due to massive bleeding during the process of RATS. It appeared to be lower than the conversion rate (11%) of patients receiving neoadjuvant chemoimmunotherapy in the CheckMate 816 trial. Therefore, RATS may be advantageous for reducing the conversion rate in stage III NSCLC based on our results.

There are several limitations in our study. First of all, this is a single-center real-world prospective cohort study, and the sample size is relatively small. Thus, the intrinsic heterogeneity of patients was unavoidable. Moreover, the single-arm design of this study does not allow us to compare the efficacy and safety of robotic and non-robotic surgical treatment stage III NSCLC, which presents an interesting line of inquiry for future studies. Second, long-term survival outcomes of these patients have not been evaluated yet. Third, we calculated the MPR and pCR rates in the total number patients who underwent surgery rather than the intention-to-treat population, which may affect the direct comparison of pathological response between other studies focusing on neoadjuvant chemoimmunotherapy and ours. However, we believe that RATS following neoadjuvant chemoimmunotherapy represents a promising therapeutic option for patients with stage III NSCLC, which requires confirmation in future randomized clinical trials.

## Conclusions

RATS following neoadjuvant chemoimmunotherapy is an effective and feasible therapeutic approach in stage III NSCLC. Patients receiving neoadjuvant chemoimmunotherapy in this study had high pathological remission rates, which is superior to neoadjuvant chemotherapy or immunotherapy alone as reported in previous clinical trials. Subsequent RATS showed a low conversion rate and low incidence of perioperative complications. More large-scale randomized studies are needed to further confirm the advantages of this therapeutic approach in stage III NSCLC.

## Data availability statement

The raw data supporting the conclusions of this article will be made available by the authors, without undue reservation.

## Ethics statement

This study was reviewed and approved by The Institutional Review Board and Ethics Committee of Central South University. The patients/participants provided their written informed consent to participate in this study.

## Author contributions

Conception and design: ZL, ML. Supervision, project administration, funding acquisition, and methodology: ZL, ML. Resources and investigation: YG, JJ, DX, YZ, HY, LW, BH, RH. Data collection: JJ, YG, YC, JZ. Data analysis: YG, JJ. Data interpretation: ML, ZL, YG, JJ; manuscript writing. All authors contributed to the article and approved the submitted version.

## Funding

This work was supported by the National Multidisciplinary Cooperative Diagnosis and Treatment Capacity Building Project for Major Diseases (Lung Cancer) of China (to ML); National Natural Science Foundation of China (grant numbers 81874327 [ZL] and 81903020 [ML]); Key Research and Development Program of Hunan Province (grant number 2019SK2251 [ZL]); and Project Program of National Clinical Research Center for Geriatric Disorders (Xiangya Hospital) (grant number 2020LNJJ02 [ZL]).

## Acknowledgments

We would like to thank the patients and families who participated in this study, as well as the healthcare teams at Xiangya Hospital, Central South University.

## Conflict of interest

The authors declare that the research was conducted in the absence of any commercial or financial relationships that could be construed as a potential conflict of interest.

## Publisher’s note

All claims expressed in this article are solely those of the authors and do not necessarily represent those of their affiliated organizations, or those of the publisher, the editors and the reviewers. Any product that may be evaluated in this article, or claim that may be made by its manufacturer, is not guaranteed or endorsed by the publisher.
