# Supplementary material for: Robotic-assisted thoracic surgery following neoadjuvant chemoimmunotherapy in patients with stage III non-small cell lung cancer: A real-world prospective cohort study
Source: Front Oncol. 2022 Aug 4;12:969545. doi: 10.3389/fonc.2022.969545 (PMC9386359; doi:10.3389/fonc.2022.969545)
Supplement: Supplementary file 1 [file Table_1.docx]

**Supplementary table 1. Detailed baseline characteristics of patients and pathological responses to neoadjuvant chemoimmunotherapy**

| Case No. | Age (y) | Gender | Smoking index | ECOG PS score | FEV1% predicted | Clinical stage | cTNM staging | Histology | Neoadjuvant therapy | Pathological response | ypTNM staging |
| --- | --- | --- | --- | --- | --- | --- | --- | --- | --- | --- | --- |
| 1 | 46 | M | 0 | 2 | 85 | ⅢB | T3N2M0 | Scc | Nivolumab+TC | pCR | T0N0M0 |
| 2 | 66 | M | 1500 | 2 | 92 | ⅢB | T4N2M0 | Scc | Nivolumab+TC | pCR | T0N0M0 |
| 3 | 35 | M | 300 | 0 | 96 | ⅢB | T4N2M0 | Asc | Nivolumab+TC | pCR | T0N0M0 |
| 4 | 64 | M | 800 | 0 | 85 | ⅢA | T3N1M0 | Scc | Nivolumab+TC | pCR | T0N0M0 |
| 5 | 58 | M | 360 | 0 | 100 | ⅢA | T2aN2M0 | Ade | Nivolumab+TC | pCR | T0N0M0 |
| 6 | 68 | M | 800 | 0 | 66 | ⅢA | T4N1M0 | Scc | Nivolumab+TC | pCR | T0N0M0 |
| 7 | 63 | M | 400 | 1 | 77 | ⅢA | T4N1M0 | Scc | Nivolumab+TC | pCR | T0N0M0 |
| 8 | 59 | M | 800 | 1 | 75 | ⅢB | T3N2M0 | Scc | Nivolumab+TP | pCR | T0N0M0 |
| 9 | 61 | M | 300 | 0 | 77 | ⅢA | T2bN2M0 | Scc | Nivolumab+TL | pCR | T0N0M0 |
| 10 | 56 | M | 600 | 0 | 88 | ⅢB | T4N2M0 | Scc | Nivolumab+TC | pCR | T0N0M0 |
| 11 | 54 | M | 0 | 0 | 85 | ⅢA | T3N1M0 | Scc | Nivolumab+TC | pCR | T0N0M0 |
| 12 | 63 | F | 0 | 1 | 75 | ⅢA | T1N2M0 | Scc | Nivolumab+TC | pCR | T0N0M0 |
| 13 | 70 | M | 0 | 1 | 68 | ⅢA | T4N0M0 | Scc | Nivolumab+TC | pCR | T0N0M0 |
| 14 | 64 | M | 800 | 1 | 89 | ⅢA | T2N2M0 | Scc | Nivolumab+TC | pCR | T0N0M0 |
| 15 | 54 | F | 0 | 0 | 95 | ⅢA | T4N0M0 | Scc | Camrelizumab+TC | pCR | T0N0M0 |
| 16 | 46 | M | 200 | 0 | 100 | ⅢB | T4N2M0 | Ade | Camrelizumab+TC | pCR | T0N0M0 |
| 17 | 64 | M | 900 | 0 | 85 | ⅢA | T2bN2M0 | Ade | Camrelizumab+PC | pCR | T0N0M0 |
| 18 | 49 | F | 0 | 0 | 90 | ⅢB | T4N2M0 | Ade | Camrelizumab+PC | pCR | T0N0M0 |
| 19 | 57 | M | 2400 | 0 | 100 | ⅢA | T2aN2M0 | Scc | Toripalimab+TC | pCR | T0N0M0 |
| 20 | 57 | M | 400 | 1 | 92 | ⅢA | T4N0M0 | Scc | Toripalimab+TC | pCR | T0N0M0 |
| 21 | 69 | M | 1000 | 2 | 60 | ⅢA | T3N1M0 | Scc | Toripalimab+TC | pCR | T0N0M0 |
| 22 | 57 | M | 800 | 1 | 89 | ⅢB | T3N2M0 | Scc | Tislelizumab+TC | pCR | T0N0M0 |
| 23 | 69 | M | 1200 | 0 | 78 | ⅢB | T4N1M0 | Scc | Tislelizumab +TC | pCR | T0N0M0 |
| 24 | 65 | M | 800 | 0 | 90 | ⅢA | T4N0M0 | Scc | Tislelizumab +TP | pCR | T0N0M0 |
| 25 | 70 | M | 2000 | 1 | 89 | ⅢA | T2aN2M0 | Scc | Sintilimab+TC | pCR | T0N0M0 |
| 26 | 46 | F | 0 | 1 | 85 | ⅢB | T3N2M0 | Scc | Pembrolizumab+TC | pCR | T0N0M0 |
| 27 | 46 | F | 0 | 1 | 96 | ⅢB | T4N2M0 | Ade | Nivolumab+TC | MPR | T1aN0M0 |
| 28 | 63 | M | 800 | 0 | 83 | ⅢB | T4N1M0 | Scc | Nivolumab+TC | MPR | T2aN0M0 |
| 29 | 68 | M | 800 | 0 | 62 | ⅢA | T4N0M0 | Scc | Nivolumab+TC | MPR | T3N0M0 |
| 30 | 70 | M | 1600 | 1 | 72 | ⅢA | T1N2M0 | Ade | Nivolumab+PC | MPR | T1bN0M0 |
| 31 | 66 | M | 600 | 1 | 68 | ⅢA | T4N1M0 | Scc | Camrelizumab+TC | MPR | T1aN0M0 |
| 32 | 69 | M | 1200 | 0 | 100 | ⅢA | T2bN2M0 | Scc | Camrelizumab+TP | MPR | T1cN0M0 |
| 33 | 64 | M | 800 | 0 | 84 | ⅢA | T3N1M0 | Ade | Camrelizumab+TP | MPR | T1cN0M0 |
| 34 | 57 | M | 1600 | 0 | 83 | ⅢB | T4N2M0 | Scc | Toripalimab+TL | MPR | T1cN1M0 |
| 35 | 61 | M | 600 | 0 | 70 | ⅢB | T4N2M0 | Scc | Sintilimab+TP | MPR | T1N0M0 |
| 36 | 58 | M | 1800 | 0 | 85 | ⅢA | T4N1M0 | Scc | Sintilimab+TP | MPR | T1N0M0 |
| 37 | 64 | M | 400 | 0 | 100 | ⅢB | T4N2M0 | Scc | Nivolumab+TC | IPR | T0N2M0 |
| 38 | 57 | M | 600 | 0 | 80 | ⅢA | T3N1M0 | Scc | Nivolumab+TC | IPR | T2aN1M0 |
| 39 | 69 | M | 0 | 1 | 66 | ⅢA | T4N0M0 | Ade | Camrelizumab+PC | IPR | T2bN0M0 |
| 40 | 62 | M | 1200 | 1 | 88 | ⅢA | T4N1M0 | Scc | Toripalimab+TC | IPR | T1cN0M0 |
| 41 | 54 | F | 0 | 0 | 75 | ⅢA | T3N1M0 | Ade | Toripalimab+PC | IPR | T2aN1M0 |
| 42 | 52 | F | 0 | 0 | 66 | ⅢB | T3N2M0 | Ade | Tislelizumab+TC | IPR | T1cN0M0 |
| 43 | 64 | M | 2500 | 0 | 75 | ⅢB | T3N2M0 | Scc | Sintilimab+TC | IPR | T2aN2M0 |
| 44 | 51 | M | 400 | 0 | 90 | ⅢA | T3N1M0 | Scc | Pembrolizumab+TC | IPR | T1N0M0 |

PS, performance status; M, male; F, female; Scc, squamous cell carcinoma; Ade, adenocarcinoma; Asc, adenosquamous carcinoma; TC, paclitaxel plus carboplatin; TP, paclitaxel plus cisplatin; PC, pemetrexed plus carboplatin; TL, paclitaxel plus lobaplatin; pCR, pathological complete response; MPR, major pathological response; IPR, incomplete pathological response; ypTNM staging, pathologic TNM stage after neoadjuvant therapy.

**Supplementary table 2. Detailed surgical outcomes of patients undergoing robotic-assisted thoracic surgery after neoadjuvant chemoimmunotherapy**

| Case No. | Extent of resection | Surgical time (min) | Estimated blood loss (mL) | Conversion to thoracotomy | Intraoperative transfusion | Complete resection | Re-surgery | Surgical complications | Postoperative length of stay (days) | 30-day mortality | 90-day mortality |
| --- | --- | --- | --- | --- | --- | --- | --- | --- | --- | --- | --- |
| 1 | Lobectomy | 235 | 100 | N | N | Y | N | N | 5 | N | N |
| 2 | Pneumonectomy | 260 | 400 | N | N | Y | N | N | 8 | N | N |
| 3 | Lobectomy | 225 | 100 | N | N | Y | N | N | 7 | N | N |
| 4 | Lobectomy | 140 | 50 | N | N | Y | N | Air leak | 21 | N | N |
| 5 | Lobectomy | 150 | 100 | N | N | Y | N | N | 4 | N | N |
| 6 | Lobectomy | 165 | 50 | N | N | Y | N | N | 8 | N | N |
| 7 | Lobectomy | 150 | 50 | N | N | Y | N | N | 5 | N | N |
| 8 | Lobectomy | 254 | 1800 | Y | RBC | Y | N | Air leak, SSI | 37 | N | N |
| 9 | Lobectomy | 175 | 50 | N | N | Y | N | N | 9 | N | N |
| 10 | Lobectomy | 180 | 200 | N | N | Y | N | N | 7 | N | N |
| 11 | Lobectomy | 220 | 50 | N | N | Y | N | Air leak | 8 | N | N |
| 12 | Lobectomy | 110 | 50 | N | N | Y | N | N | 4 | N | N |
| 13 | Lobectomy | 200 | 50 | N | N | Y | N | Chylothorax | 15 | N | N |
| 14 | Lobectomy | 275 | 150 | N | N | Y | N | N | 8 | N | N |
| 15 | Lobectomy | 75 | 50 | N | N | Y | N | N | 4 | N | N |
| 16 | Lobectomy | 130 | 20 | N | N | Y | N | N | 7 | N | N |
| 17 | Lobectomy | 250 | 500 | N | RBC | Y | N | N | 5 | N | N |
| 18 | Bilobectomy | 205 | 100 | N | N | Y | N | N | 3 | N | N |
| 19 | Lobectomy | 190 | 100 | N | N | Y | N | N | 5 | N | N |
| 20 | Lobectomy | 250 | 100 | N | N | Y | N | N | 6 | N | N |
| 21 | Lobectomy | 174 | 100 | N | N | Y | N | N | 5 | N | N |
| 22 | Lobectomy | 175 | 50 | N | N | Y | N | Chylothorax | 12 | N | N |
| 23 | Lobectomy | 110 | 50 | N | N | Y | N | N | 11 | N | N |
| 24 | Lobectomy | 230 | 500 | N | RBC | Y | N | N | 12 | N | N |
| 25 | Lobectomy | 280 | 200 | N | N | Y | N | N | 7 | N | N |
| 26 | Lobectomy | 95 | 50 | N | N | Y | N | N | 5 | N | N |
| 27 | Lobectomy | 148 | 100 | N | N | Y | N | N | 6 | N | N |
| 28 | Lobectomy | 120 | 100 | N | N | Y | N | N | 5 | N | N |
| 29 | Lobectomy | 195 | 50 | N | N | Y | N | N | 5 | N | N |
| 30 | Lobectomy | 210 | 100 | N | N | Y | N | N | 4 | N | N |
| 31 | Lobectomy | 170 | 100 | N | N | Y | N | N | 4 | N | N |
| 32 | Lobectomy | 155 | 50 | N | N | Y | N | N | 11 | N | N |
| 33 | Lobectomy | 315 | 200 | N | N | Y | N | N | 12 | N | N |
| 34 | Bilobectomy | 196 | 200 | N | N | Y | N | N | 7 | N | N |
| 35 | Sleeve lobectomy | 320 | 600 | Y | N | Y | N | N | 10 | N | N |
| 36 | Lobectomy | 260 | 500 | N | N | Y | N | N | 6 | N | N |
| 37 | Lobectomy | 270 | 500 | N | N | Y | N | N | 6 | N | N |
| 38 | Lobectomy | 230 | 30 | N | N | Y | N | N | 7 | N | N |
| 39 | Lobectomy | 122 | 50 | N | N | Y | N | N | 5 | N | N |
| 40 | Sleeve lobectomy | 305 | 100 | N | N | Y | N | N | 6 | N | N |
| 41 | Lobectomy | 190 | 50 | N | N | Y | N | N | 7 | N | N |
| 42 | Lobectomy | 192 | 100 | N | N | Y | N | N | 5 | N | N |
| 43 | Lobectomy | 190 | 50 | N | N | Y | N | N | 9 | N | N |
| 44 | Lobectomy | 190 | 50 | N | N | Y | N | N | 5 | N | N |

N, no; Y, yes; RBC, red blood cells; SSI, surgical site infection.
